# Hypoxia increases triacylglycerol levels and unsaturation in tomato roots

**DOI:** 10.1186/s12870-024-05578-4

**Published:** 2024-09-30

**Authors:** Johanna Striesow, Marcel Welle, Larissa Milena Busch, Sander Bekeschus, Kristian Wende, Christine Stöhr

**Affiliations:** 1 ZIK plasmatis, Leibniz Institute for Plasma Science and Technology (INP), Felix-Hausdorff-Str. 2, 17489 Greifswald, Germany; 2 Institute of Botany & Landscape Ecology, Greifswald University, Soldmannstr. 15, 17489 Greifswald, Germany; 3 Department of Functional Genomics, Greifswald University Medical Center, Felix-Hausdorff- Str. 8, 17489 Greifswald, Germany

**Keywords:** Lipidomics, Mass spectrometry, Plant stress, Stress response

## Abstract

**Background:**

Plants are designed to endure stress, but increasingly extreme weather events are testing the limits. Events like flooding result in submergence of plant organs, triggering an energy crisis due to hypoxia and threaten plant growth and productivity. Lipids are relevant as building blocks and energy vault and are substantially intertwined with primary metabolism, making them an ideal readout for plant stress.

**Results:**

By high resolution mass spectrometry, a distinct, hypoxia-related lipid composition of *Solanum lycopersicum* root tissue was observed. Out of 491 lipid species, 11 were exclusively detected in this condition. Among the lipid classes observed, glycerolipids and glycerophospholipids dominated by far (78%). Differences between the lipidomic profiles of both analyzed conditions were significantly driven by changes in the abundance of triacylglycerols (TGs) whereas sitosterol esters, digalactosyldiacylglycerols, and phosphatidylcholine play a significantly negligible role in separation. Alongside, an increased level of polyunsaturation was observed in the fatty acid chains, with 18:2 and 18:3 residues showing a significant increase. Of note, hexadecatetraenoic acid (16:4) was identified in hypoxia condition samples. Changes in gene expression of enzymes related to lipid metabolism corroborate the above findings.

**Conclusion:**

To our knowledge, this is the first report on a hypoxia-induced increase in TG content in tomato root tissue, closing a knowledge gap in TG abiotic stress response. The results suggest that the increase in TGs and TG polyunsaturation degree are common features of hypoxic response in plant roots.

**Supplementary Information:**

The online version contains supplementary material available at 10.1186/s12870-024-05578-4.

## Introduction

The sessile lifestyle forces plants to cope with different stresses during their lifecycle. With increased extreme weather events, the impact on plant growth and productivity becomes more severe [1, 2]. Floods and heavy rainfalls result in waterlogging and submergence of plant organs reducing gas exchange rates. The decline in gas diffusion under these conditions limits oxygen availability and causes an energy crisis due to hypoxia. Therefore, plants evolved several metabolic and morphological adaptations. Energy production is shifted to anaerobic fermentation, and aerenchyma formation facilitates gas transport within the plant [3]. In addition to the toxic metabolites ethanol and lactate, nitric oxide and reactive oxygen levels are highly increased under hypoxia [4].

Lipids are crucial for membrane integrity or energy metabolism and are usually well controlled, e.g. to maintain cell membrane functionality [5]. Abiotic stress factors trigger adaptive responses that have been reported to result in alterations of the lipid composition [6]. Several recent studies highlight an association between increased triacylglycerol (TG) levels and abiotic stress [7, 8]. These storage lipids are normally found in seeds and fruits. They accumulate in vegetative tissue in lipid droplets under stress. A protective function of the droplets itself or by the sequestration of toxic lipid intermediates has been considered [9, 10].

Nevertheless, little is known about the role of TGs and alterations in the fatty acid composition in the response towards hypoxia [11]. Besides their role in membrane fluidity control, polyunsaturated fatty acids are stress hormone precursors and may reveal regulatory activity in stress response [12, 13]. Recent findings indicate the importance of lipids and fatty acids in oxygen sensing. Unsaturated long-chain Acyl-CoAs modulate Ethylene Responsive Factor VII (ERF VII), a key player in hypoxic response in plants [14–16]. Unsaturated very-long-chain ceramide species modulate the ethylene signaling pathway under hypoxia [14]. A closer look at low-abundant and stress-specific lipids can increase our understanding of plant stress adaptation.

Being the first organ to experience changes in oxygen availability, the root physiology is important to understand adaptation processes. Therefore, a hypoxia model based on the partial submergence of tomato plants was used to collect root material. Subsequently, lipids were extracted and analyzed by a bottom-up shotgun lipidomics approach using ultra-high performance liquid chromatography coupled to a high-resolution tandem mass spectrometer (UHPLC-MS^2^). The study provides valuable information about the impact of hypoxic stress on root tissue lipid composition, with a special focus on TG levels and the degree of saturation of their fatty acid chains.

## Materials and methods

### Chemicals

Chloroform was of HPLC grade and obtained from Roth. Isopropanol, acetonitrile and methanol had LC-MS grade; water had HPLC grade. Those chemicals were obtained from Th.Geyer. Formic acid was purchased from Honeywell and had LC-MS grade. Ammonium formate was obtained from Sigma-Aldrich and had LC-MS grade. EquiSPLASH Lipidomix was purchased from Avanti Polar Lipids. Magnesium sulfate, calcium nitrate and calcium sulfate were obtained from AppliChem. Other chemicals needed for plant growth were obtained from Merck.

### Cultivation of Solanum lycopersicum under root hypoxia and harvest

*Solanum lycopersicum* (L.) *cv*. Moneymaker was cultivated under greenhouse conditions (14 h light 18 °C / 10 h dark 22 °C). Tomato plants grew on quartz sand culture for four weeks with a nutrient solution containing 5 mM NO_3_^-^ [17, 18]. Tomato roots were waterlogged for 48 h to apply hypoxic conditions. Therefore, trays were filled with distilled water until the quartz sand substrate was covered. Prior to harvest, the quartz sand mixture was carefully removed from plants using ice-cold water. Two sets of plants were independently grown. Nine control and nine waterlogged plants were harvested per set and prepared separately (*N* = 18 biological replicates).

### Lipid extraction

Lipids from tomato roots were extracted with a modified procedure adapted from Shiva and colleagues [19]. In brief, 0.1 g of grinded roots (cooled on liquid nitrogen) were added to a glass reaction tube filled with isopropanol supplemented with 0.01% BHT. The mixture was incubated for 15 min at 75 °C and cooled on ice afterward. To the cooled-down mixture, 0.5 volume of chloroform, 0.2 volume of water and 3 µL of EquiSPLASH Lipidomix were added. Lipids were extracted for 1 h on ice with occasional vortexing in between. Reaction tubes were centrifuged at 5000 x g to induce phase separation, and the organic phase was transferred to another glass reaction tube. To the remaining water phase, 1.33 volumes of a chloroform: methanol mixture (2:1, v/v) supplemented with 0.1% BHT was added and incubated for 30 min on ice with occasional vortexing. Reaction tubes were again centrifuged at 5000 rpm, and the chloroform phase was transferred to the second glass vial. The chloroform: methanol extraction step was repeated with the remaining water phase 3 times. Afterward, 0.33 volumes of KCl (1 M) were added to the combined lipid extracts and vortexed. The upper water phase was removed, and 0.66 volumes of water were added and vortexed. The upper phase was again removed, and sodium sulfate was added to remove the remaining water. Finally, the lipid extract was dried under constant N_2_-flow with a TurboVap sample evaporator (Biotage, Sweden) and frozen at -80 °C until MS analysis.

### Reversed-phase LC-MS2

Frozen lipid films were rehydrated with chloroform: methanol: isopropanol (1:2:4 v/v/v, supplemented with 5 mM ammonium formate). Lipids were separated on a Vanquish UHPLC equipped with an AccuCore C30 column and guard column (150/10 × 2.1 mm, Thermo Fisher) at 50 °C. Mobile phases were acetonitrile/water (60:40 v/v, A) and isopropanol/acetonitrile (90:10 v/v, B), both supplemented with 10 mM ammonium formate and 0.1% formic acid. At a flow rate of 350 µL/min the following gradient was used: 0–3 min 30% B, 3–8 min 50% B, 8–22 min 70% B, 22–29 min 99% B, 29–37 min hold 99% B, 37–37.1 min decrease to 30% B, 37.1–41 min hold 30% B. The LC was connected to a QExactive Plus mass spectrometer (Thermo Fisher), operated in data dependent acquisition mode (top 15) with a dynamic exclusion time of 10 ms. Samples were were injected four times (2x positive and 2x negative mode, two technical replicates per biological replicate). For the full MS survey scans, a range of 100 to 1000 m/z was set and the automatic gain control (AGC) target was 1 × 10^6^ with a maximum ion time (IT) of 80 ms. Resolution used in full MS was 70.000, for MS/MS after higher-energy collisional dissociation 17.500 (HCD, each at 200 m/z). HCD was achieved with a stepped normalized collision energy from 25 to 27 in positive mode and from 20 to 23 in negative mode with an AGC target of 2 × 10^5^ and a maximum IT of 50 ms.

### Identification of lipids

MS raw files were analysed with the software LipidSearch (version 4.2.27, Thermo Fisher) using the following parameters: retention time interval 0.01 min, m-score threshold 5, precursor tolerance 5 ppm, product tolerance 8 ppm, ID quality filter A (fatty acid chain and class identified completely) and B (class and some fatty acid chains identified). The resulting text files were processed with an in-house KNIME workflow and filtered for ppm error, peak quality and area score. The area of identical lipids with different ion adducts was summed up. The resulting excel file was processed in R (v 4.0.2) with manual inspection of raw areas (Figure S1A). Lipids in the blanks or blank extracts were excluded from the data, if not at least 5-fold lower in intensity in blank controls. Lipid areas were normalized to the respective lipids in the EquiSplash standard, and median normalization was applied (Figure S1B). Lipids were filtered for entries, that had to be present in both sets and in at least 50% of the samples within at least one condition. For principal component analyses (PCA), lipid species identified in at least 50% of samples were considered, and the two independent sets were batch-adjusted using the ComBat algorithm. Lipids were annotated on the molecular species level in accordance with the proposed nomenclature by the LIPID MAPS consortium since no information on the sn-position of acyl/alkyl constituents was available [20, 21].

### Transcriptome analysis

Transcriptomic raw data (deposited at Sequence Read Archive (SRA) database (bioproject accession PRJNA553994), obtained by NextSeq. 500 Illumina platform (LGC Biosearch Technologies) and processed by CLC Genomics Workbench Software (Qiagen, V. 7.5.5) and published by Safavi-Rizi and colleagues (2020), were reevaluated as described in the same paper with a focus on lipid-related transcriptomic changes [22].

### Statistical analysis

To analyze lipid classes contributing to the separation of experimental conditions assessed via PCA, Fisher’s Exact test was used. Lipid species contributing more than two-fold the expected weight to the particular dimension were considered to span the dimension. For statistical analysis of TGs, data were tested for normal distribution with the Shapiro-Wilk test and subsequently tested for statistical significance with 2-way ANOVA with Sidak post-hoc test or multiple t-test with Holm-Sidak post-hoc test (tested against control, *** *p* < 0.001; ** *p* < 0.01, * *p* < 0.05).

## Results

### Hypoxia alters the tomato root lipidome composition significantly

**Fig. 1:**
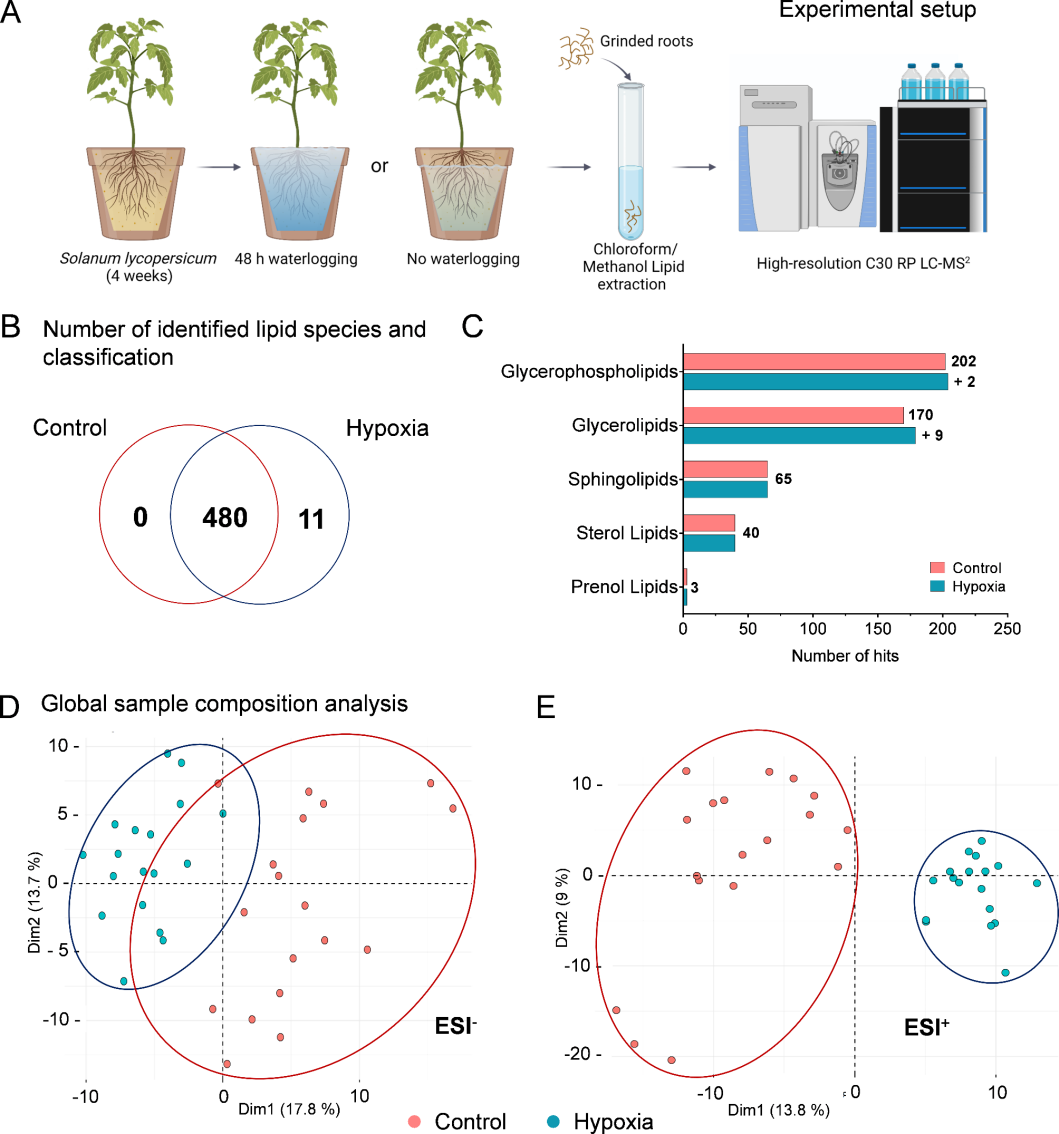
Experimental workflow and the root lipidome variation under hypoxic conditions. Tomato plants grew for 4 weeks on quartz sand culture under greenhouse conditions before they were waterlogged for 48 h, harvested and grinded in liquid nitrogen. After chloroform/methanol extraction, lipids were analyzed with UHPLC-MS^2^ (**A**). The detected root lipidome of waterlogged or control tomato plants consisted of 480 lipid species, 11 species were exclusively identified under hypoxic conditions (**B**). The majority of the detected lipid species belonged to five lipid classes, with glycerol- and glycerophospholipids being the largest group. Hypoxic conditions increased the number of glycerol- and glycerophospholipid species (**C**). PCA-plots of the first and second dimension calculated based on the 50 percentile lipidome profiles of the samples, which show a separation of samples belonging to the control and hypoxia condition groups in negative (**D**) and positive polarity (**E**) along the first dimension

Little is known about the plant lipidome and its adaptation to hypoxic stress. Therefore, the lipid composition of *Solanum lycopersicum* roots after 48 h of waterlogging was analyzed by high resolution mass spectrometry **(**Fig. 1A**)**. With this approach, a total of 491 lipid species were identified. The majority (480) of lipid species were detected under both control and hypoxic conditions, while 11 were exclusively found under hypoxia **(**Fig. 1B**)**. Lipids belonging to five categories were detected, with glycerophospholipids and glycerolipids dominating. Together, they comprised 78% of all identified lipids. Besides, minor amounts of sphingo-, sterol and prenol lipids were found. The lipids exclusively found under hypoxia were among the most frequently detected categories − 9 were glycerolipids, and 2 were glycerophospholipids (Fig. 1C). While all new glycerolipids belonged to the class of TGs, the two glycerophospholipids were phosphatidylethanolamines (PE). Interestingly, most fatty acid chains of newly identified TG species showed a high degree of unsaturation (Table S1). A principal component analysis (PCA) revealed an altered lipidomic profile under hypoxic conditions in both polarities (Fig. 1D, E). The negative and positive polarity of the first two principal components/ dimensions of the PCAs accounted to 31.5% (17.8% and 13.7%) and 22.8% (13.8% and 9%) of the total lipidomic profile variances, respectively, pointing to interindividual sample specific lipidomic profiles. Despite modest variance explanatory power, the control and hypoxia samples were clearly separated along the first dimension. This leads to the suggestion that the hypoxia and control conditions applied to the samples are the main drivers for differences in the lipidomic profiles. Subsequently, to analyze the main condition-separating differences in the lipidomic profiles Fisher’s Exatct test was used. Enrichment or reduction of lipid class dimension was indicating that TGs are significantly enriched, whereas phosphatidylcholine, digalactosyldiacylgylcerol (DGDG) and acyl hexosyl sitosterol esters were reduced in their importance in the separation of the conditions (Table S2).

### Triacylglycerol metabolism is affected under hypoxic conditions

**Fig. 2:**
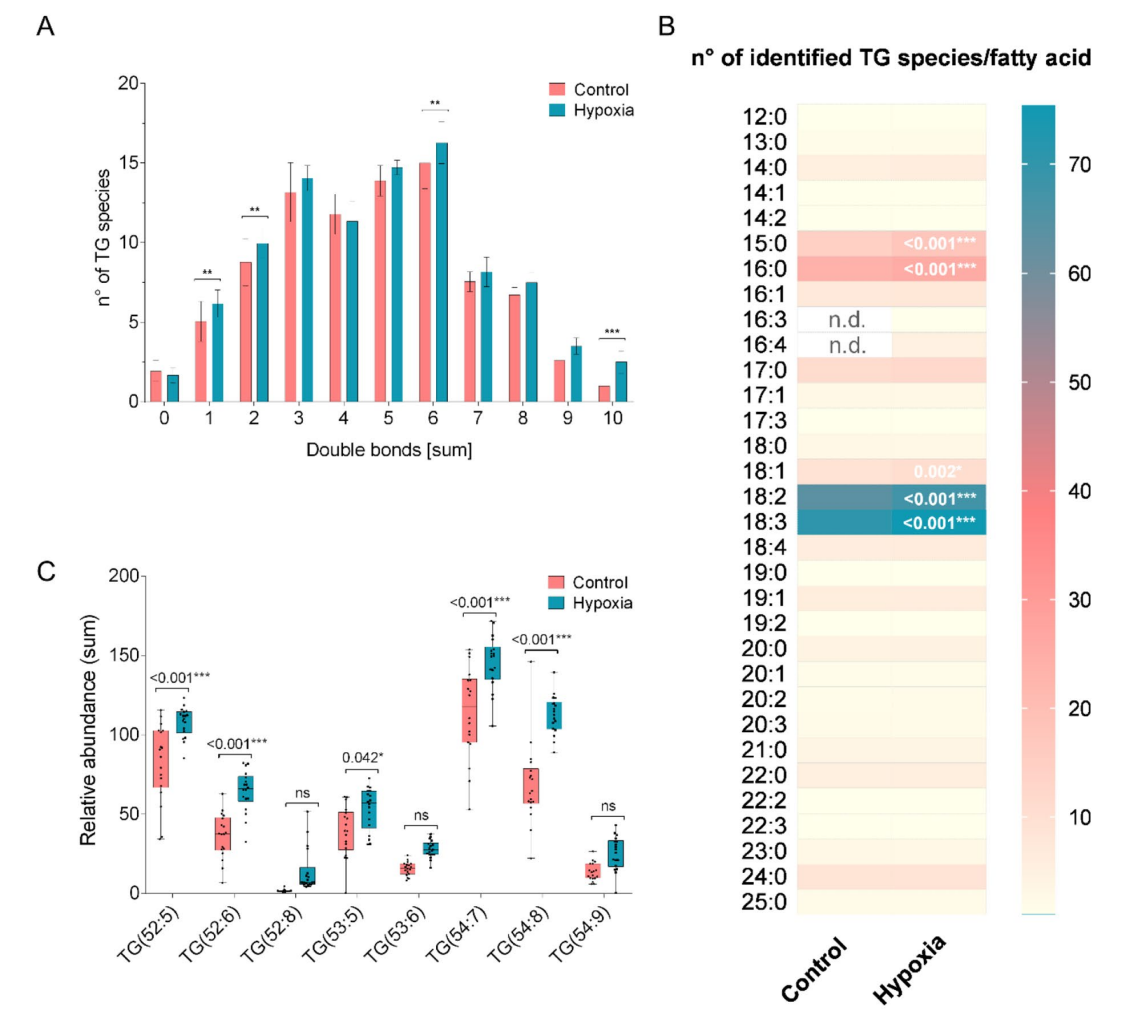
TGs increase their double bonds and the number of species esterified with 15:0, 16:0, 18:1, 18:2, and/or 18:3 fatty acids during hypoxic conditions. Absolute number of TG species were plotted against their sum of double bonds for control and hypoxic samples (**A**). The number of species esterified with 15:0, 16:0, 18:1 18:2, and/or 18:3 fatty acids is significantly increased under hypoxic conditions (**B**). TG species with significant change in abundance under hypoxic conditions compared to control, insert shows lower abundant species (**C**). Data show mean and S.D.; statistical analysis was performed using multiple t-testing with Holm-Sidak post-hoc test (tested against control, *p ≤ 0.05, **p ≤ 0.01, ***p ≤ 0.001)

Since TG species were shown [9, 10] to carry information about lipidome separation of the analyzed conditions and most newly detected lipids were TGs, this class underwent further analysis. First, changes in saturation degree were evaluated. Under hypoxic conditions, an increased number of unsaturated TG species was observed while the number of saturated TGs decreased. TG species with one to ten double bonds were increased in number, significant for species with one, two, six and ten double bonds per molecule (Fig. 2A). In addition to the TG count, the abundance of TG bulk species underwent further analysis. Most abundant TG bulk species across both conditions were 54:7 and 54:8 TGs, while low abundancies were observed for TGs with shorter fatty acid chains (TG 52:5/6 and 53:5). In case of hypoxic conditions, five TG bulk species were significantly increased. Interestingly, no TG species was more abundant under control than hypoxic conditions (Fig. 2C). Under hypoxic conditions, it was observed that TG species incorporating fatty acids with a higher degree of unsaturation were both more versatile (higher number of identified species) and showed a higher abundancy than the more saturated counterparts. A detailed look at the fatty acid profile may provide information on TG remodeling and origin during hypoxic stress conditions at the root level. Since bulk species abundance does not carry information on individual esterified fatty acids, TG species were categorized and counted in relation to their double bonds. In general, hypoxic samples were richer in fatty acid species. The four most prevalent fatty acid species were 18:2, 18:3, 15:0 and 16:0, all of them component of a higher number of TG species under hypoxia. Interestingly, the fatty acids 16:3 and 16:4 were exclusively detected in new identified TGs (Fig. 2B). In total, nine TGs were identified that were not present in any of the control plants (Table S1**)**. Since the detected compounds carry an unusual fatty acid (hexadecatetraenoic acid, 16:4), they were manually inspected for correct MSMS fragmentation pattern and peak shape (Figure S2). Interestingly, also two PE species with the unusual 16:4 fatty acids were identified and manual inspected (Figure S4).

### The abundance of TG species with 16:0, 18:2 and/or 18:3 fatty acids increase under hypoxia while the abundance of the TG-precursors PA, DG, LPC and MGDG decrease

**Fig. 3:**
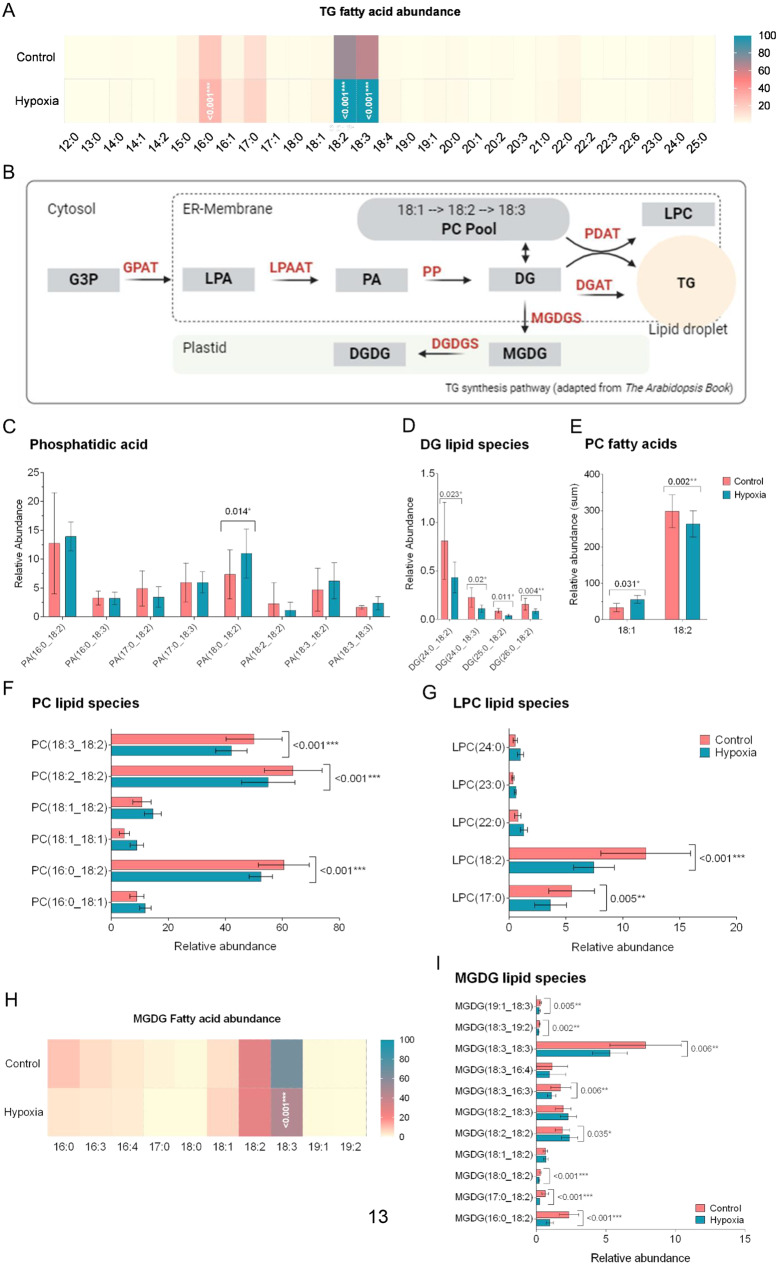
The abundance of 16:0, 18:2 and 18:3 esterified TG species increases while some matching precursor PA, DG, LPC and MGDG decrease significantly under hypoxic conditions. Comparison of TG species (sum of all identified in both conditions) reveals increase in abundance in species incorporating 16:0, 17:0, 18:2 or 18:3 fatty acids (**A**) Pathway of TG synthesis. (adapted from Li-Beisson et al.,* 2013*) (**B**). Comparison of TG synthesis precursors phosphatidic acid identified in both conditions (**C**), Significantly changed DG lipid species (24:0_18:3 and DG 26:0_18:3, **D**), PC (sum of 18:1 and 18:2 esterified species, **E** and individual lipid species, **F**), LPC (**G**) and MGDG (abundance of fatty acids (**H**) and individual lipid species (**I**)). Data show mean and S.D.; statistical analysis was performed using multiple t-testing with Holm-Sidak post-hoc test (*p ≤ 0.05, **p ≤ 0.01, ***p ≤ 0.001)

Beside the number of TG species (Fig. 2C), also the abundance of TG species esterified with 16:0, 18:2 and 18:3 fatty acids were significantly increased (Fig. 3A). Other long-chain unsaturated fatty acids incorporating TGs (18:1, 18:4) and 17:0 fatty acids showed a trend to higher abundance (Fig. 3A). The 16:0, 18:2 and 18:3 fatty acids introduced into the newly formed TGs in stress conditions may derive from *de-novo* synthesis or have been diverted from the synthesis of other lipid species. Precursors of TG can result from the Kennedy pathway in the ER membrane from conversion of phosphatidic acid (PA) to DG. TG is then formed from diacylglycerol (DG) by diacylglycerol acyltransferase. Another route of TG formation is the phosphatidylcholine (PC) pool as source of acyl species, catalyzed by phosphatidylcholine acyltransferase (PDAT). As further product lysophosphatidylcholine (LPC) is formed (Fig. 3B). For PA, an increase in abundance under hypoxia was observed (Fig. 3C). Interestingly, two PA species with an unusual fatty acid composition accumulated in waterlogged plant roots (Figure S4A). According to the MSMS fragmentation spectra (Figures S4D-G), both species were esterified with16:4 fatty acids. Out of the detected DGs the DG species 24:0_18:2, 24:0_18:3, 25:0_18:2 and 26:0_18:2 were significantly altered. All were reduced in abundance under hypoxic conditions (Fig. 3D). The abundance of PC species was decreased in hypoxic conditions. Out of the six detected PC species 3 were significantly decreased in abundance. Higher levels of PC 18:1_18:2, 18:1_18:2 and 16:0_18:1 were observed under control conditions (Fig. 3F). With a look on fatty acids species in PC lipids, 18:1 was more abundant while 18:2 was less abundant under hypoxic conditions (Fig. 3E). The other fatty acids remained unchanged. Together with PC, LPC species significantly decreased with LPC 17:0 and LPC 18:2 been significantly altered (Fig. 3G). In accordance with the literature, fatty acid precursors for TG biosynthesis in vegetative tissue may derive from monogalactosyldiacylglycerol (MGDG) and are transferred via PC yielding TGs, catalyzed by PDAT [23]. Corroborating this, the abundance of MGDG species esterified with an 18:3 fatty acid is significantly reduced, and for the fatty acids 16:0 and 16:3 a trend towards a reduced abundance was observed (Fig. 3H). For individual MGDG species, a reduction was detected for compounds with an 18:2 or 18:3 fatty acid (four and three lipid species, respectively). Only MGDG 18:2_18:2 showed higher abundancy under hypoxia. In addition, the abundance of one MGDG species carrying a 16:3 fatty acid was reduced. The MDGD species carrying 16:4 fatty acid were undisturbed by the hypoxia (Fig. 3I). In plastids, MGDG are metabolized to DGDGs. Under the current conditions, no changes were detected for this lipid class (Figure S3). These findings indicate that especially 18:2 and 18:3 fatty acids accumulating in TGs may derive from MGDGs via the PC pool and LPCs formation by DG integration.

Safavi-Rizi and colleagues investigated the effects of 48 h hypoxia on the transcriptome of tomato roots (Sequence Read Archive database, bioproject accession PRJNA553994) [22]. The raw datasets also contained information on lipid metabolism, which was not addressed so far and therefore were reanalyzed in the frame of this study. Among the five most upregulated genes, two ACB-desaturases, OLEOSIN1, a lysophospholipase and a DGDG synthase (DGD2) were identified (Table 1**)**. Strong down-regulation was observed for a DGDG synthase (DGD1), a lipid transfer protein (LP1), an acyl-CoA carboxylase, PMEAMT – an enzyme involved in choline and phospholipid synthesis [24] – and a TG lipase (Table 2).

In summary, newly formed TGs incorporate more likely, 18:2 and 18:3 fatty acids in their molecule while at the same time also abundance of preexisting species carrying the same fatty acids were decreased.

**Table 1:** Up-regulated gene expression in the biological category *lipid metabolism* after 48 h of water logging (fold change)

Identifier	Biological category	Arabidopsis homolog	Fold change
solyc11g008680.1.1	FA synthesis and FA elongation; ACP desaturase	AT2G43710.2: SSI2, FAB2, Plant stearoyl-acyl-carrier-protein, desaturase (family)	+ 55.1
solyc12g010920.1.1	TAG synthesis	AT4G25140.1, OLEO1, OLE1, oleosin1	+ 43.9
solyc12g042890.1.1	lipid degradation, lysophospholipases, carboxylesterase	AT1G52700.1: α/β-Hydrolase	+ 39.0
solyc01g094170.2.1	glycolipid synthesis, DGDG synthase	AT4G00550.1: DGD2, digalactosyl diacylglycerol deficient 2	+ 10.4
solyc06g053480.2.1	FA synthesis and FA elongation, ACP desaturase	AT2G43710.2: SSI2, FAB2, Plant stearoyl-acyl-carrier-protein, desaturase (family)	+ 7.6
solyc02g082910.2.1	FA synthesis and FA elongation, acyl coa ligase	AT1G20560.1: AAE1, Acyl activating enzyme 1	+ 6.2
solyc09g008840.2.1	FA synthesis and FA elongation.pyruvate kinase	AT3G52990.1: Pyruvate kinase family protein	+ 5.6

**Table 2:** Down-regulated gene expression in the biological category *lipid metabolism* after 48 h of water logging (fold change)

Identifier	Biological category	Arabidopsis homolog	Fold change
solyc01g007100.2.1	glycolipid synthesis, DGDG synthase	AT3G11670.1: DGD1, UDP-Glycosyltransferase	- 21.4
solyc08g067500.1.1	lipid transfer proteins	AT2G38540.1: LP1, LTP1, ATLTP1, lipid transfer protein 1	- 19.7
solyc03g025720.2.1	FA synthesis and FA elongation, acyl CoA ligase	AT3G48990.1: AMP-dependent synthetase and ligase	- 9.9
solyc12g040790.1.1	Phospholipid synthesis	AT1G48600.2: PMEAMT, AtPMEAMT, S-adenosyl-L-methionine-dependent methyltransferase	- 9.3
solyc02g076990.2.1	lipid degradation, lipases, triacylglycerol lipase	AT4G18550.1: α/β-Hydrolase	- 5.7
solyc05g011860.1.1	‘exotics’ (steroids, squalene)	AT2G03760.1: ST, ATST1, RAR047, ST1, AtSOT1, AtSOT12, SOT12 sulphotransferase 12	- 2.9

## Discussion

By inducing hypoxic conditions in plant roots, a broad stress response is triggered for handling this detrimental condition. Changes in carbon metabolism – including lipid metabolism, signal processes, and anatomical adaptations were found prominent [22, 25]. The alteration in lipid composition is a known feature of stress adaptation. It ensures membrane integrity and energy resources [26], but also the sensing of stress conditions and the subsequent activation of downstream processes [27]. For hypoxia, limited data are available for *Arabidopsis*, where changes in glycerophospholipids, galactolipids, and ceramides were found [14, 28]. The data presented here indicate that hypoxia significantly altered the lipid composition of tomato roots and led to a hypoxia-specific pattern. While phosphatidylcholine (PC) lipid levels decreased in hypoxic tomato roots (Fig. 3C), confirming observations in *A. thaliana* rosette and leaf [14, 28], other glycerophospholipids and ceramides remained unaltered. In contrast, TGs were found to contribute most prominently to differences in the lipidome under hypoxic conditions (Figure S2). This observation is in agreement with other studies linking TG accumulation in vegetative tissue to abiotic stress [10]. Under drought, heat, cold, and nutrient starvation, an increase in TG content was observed [29, 30].

In vegetative tissue, *de novo* synthesized diacylglycerol (DG) is acylated into TG via diacylglycerol acyltransferase (DGAT) or phospholipid: diacylglycerol acyltransferase (PDAT) (reviewed by [10]). Heat stress led to releasing free fatty acids (FFAs) from MGDG. PDAT used FFAs converted into PC to build TGs [31–33]. Under freezing and water deficiency conditions, DAG derived from MGDG or DGDG and trigalactosyldiacylglycerol (TGDG), respectively. DGAT used the DAG to form TG [30, 34, 35]. Under ozone stress, FFAs and DAG for TG synthesis are derived from MGDG [36, 37]. In this study on hypoxic tomato roots, the content of TG species and their total abundance increased while PC, LPC, DG and MGDG abundance decreased (Fig. 2 + 3). The increase in TGs and decrease in PC and MGDG could indicate a similar sensing or management mechanism as under heat stress and point to a role in general stress coping mechanism for TGs.

Assessment of the mRNA profile revealed distinct changes due to the hypoxic conditions. The induction of desaturases in stress conditions is consistent with the increase in unsaturated fatty acids (Fig. 2A). The upregulation of OLEOSIN1, a structural lipid body protein [38], and the reduction in gene expression of a TG lipase may indicate TG accumulation in lipid bodies. The induction of DGD2 but the reduction of DGD1 transcription fits with the functional descriptions of Kelly and Dörmann according to which DGD1 is the major DGDG synthase and DGD2 is active under stress conditions [39]. This is in accordance with observed unaltered DGDG abundance (Figure S3) and less importance of DGDGs for differentiation of control and hypoxic lipid profile in the enrichment analysis (Table S2). The reduction of PC and LPC (Fig. 3C + G) are also detectable in the transcriptomic data.

The role of accumulated TGs – generally considered as storage lipids – under stress conditions is controversially discussed in the scientific community. One explanation may be the segregation of toxic lipid intermediates. During the adaptational response to abiotic stress, changes in lipid composition ensure membrane integrity [26]. The toxic intermediates DAGs and FFAs are released from thylakoid membranes during this process. As a result of this, TG synthesis might reduce their concentration and attenuating negative effects [40]. In *Arabidopsis*, the key player phytyl ester synthase 1 and 2 (PES1 and 2) involved in this process are induced under nitrogen starvation and senescence [41]. Additionally, the importance of TGs in lipid droplets as a scaffold for several stress-responsive proteins was highlighted [42]. Best characterized is caleosin, an abundant lipid droplet-associated protein involved in oxylipins and phytoalexins production [42–45]. OLEOSIN1 is the main protein on lipid body surface and involved in their structural integrity [46]. Interestingly, gene expression of this protein is upregulated upon 48 h hypoxia in tomato roots (Tabel 1). The mutation of lipid droplet-associated small rubber particle proteins (SRPs) reduced drought tolerance and emphasized the importance of lipid droplets in stress response [45]. Due to the ubiquitous appearance of TG accumulation in vegetative tissue under stress conditions, a similar function of TG under hypoxia is likely.

The hypoxia-relevant TG species showed an increased degree of unsaturation, and higher numbers of double bounds were favored. Unsaturation of membrane lipids plays an important role in response to different stressors such as chilling, freezing, heating, salinity and drought [47–52]. Hypoxia increased fatty acid unsaturation in both *Arabidopsis* leaves and crown galls via an increased expression of desaturases [14, 53]. A recent study even highlighted the importance of polyunsaturated linolenoyl-CoA (18:3-CoA) modulating hypoxic response in *A. thaliana* [16]. Under heat stress, 18:3 is removed from thylakoid-derived MGDG and stored in TGs to ensure membrane integrity [33]. An increase in 18:3 fatty acids had also been observed in hypoxic Arabidopsis crown galls [53]. The current study showed, that the number TG species with 18:2 and 18:3 fatty acids and their total abundance increased in hypoxic tomato roots compared to normoxic controls (Fig. 3A**)**. Those fatty acids are known to be enriched in MDGDs and DGDGs [54]. In this study, the origin of the polyunsaturated fatty acids could not be fully solved. Reduced MGDG levels and abundant polyunsaturated C-18 fatty acids (Fig. 3I) point to thylakoidal origin from galactosylglycerols, alongside with the PC pool as a central fatty acid buffer. A role of TGs in free fatty acid sequestration is likely.

Notably, several polyunsaturated PE and TG lipid species were exclusively identified under hypoxic conditions. All incorporated a 16:4 fatty acid (Figure S1) similar to stearidonic acid (ω-3 18:4). TG (16:3_16:4_18:2) additionally showed an unusual 16:3 fatty acid (Figure S1). A database search revealed that those lipid species were not included in the general LIPID MAPS and other databases. Additionally, they were not described as part of the plant lipidome of *Solanum lycopersicum* until now. Since no commercial standard for those compounds is available, information on specific MSMS fragmentation behavior is unknown. At large, 16:4 fatty acids were considered to occur in pacific krill [55] but mainly in algae species [56] and are used as a taxonomic indicator for algae classes [57]. So far, only a few reports show its presence in higher plants [58–61]. Findings of 16:4 fatty acids in tomato fruit skin are reported [62, 63]. Besides, there are reports of dinor-oxo-phytodienoic acid (dnOPDA, 16:4-O) with respect to MGDG/DGDG acylation and stress response [64–66]. 16:3 fatty acids accumulated in TG species in an *Arabidopsis sdp1* mutant (TG lipase) and was reported to be of MGDG/DGDG origin [67, 68]. In addition, MGDG/DGDG 16:3 fatty acids are linked to several stressors [69, 70]. Our study also identified MGDG containing both 16:3 or 16:4 fatty acids **(**Fig. 3H + I). The appearance of this unusual TG and PE species containing 16:4 and/or 16:3 fatty acids in tomato roots seemed to be stress induced. An origin of the fatty acids from MGDG could be possible. In terms of stress induced TG accumulation, *Chlamydomonas reinhardtii* 16:4 fatty acids show a similar role as 18:3 fatty acids in higher plants. A relict regulatory function of these hypoxia induced fatty acid would be thinkable.

## Conclusion

Hypoxia induced by partial submergence of tomato plants boosts TG content and increases fatty acid polyunsaturation in root tissue, thereby resembling features described for other types of plant stress. The results suggest that the increase in TGs and TG polyunsaturation degree are common features of hypoxic response in plant roots. It may be assumed that those TGs are implemented in lipid droplets which are known to be involved in sequestration toxic lipid intermediates. To our knowledge, this is the first report on a hypoxia-induced increase in TG content in tomato root tissue, closing a knowledge gap in TG abiotic stress response.

## Electronic supplementary material

Below is the link to the electronic supplementary material.

Supplementary Material 1

## Data Availability

The underlying data of this manuscript were deposited at www.metabolomicsworkbench.org under study ID ST002880.

## Abbreviations

AcHexSiE: Acyl hexosyl sitosterol ester
DG: Diacylglyceride
DGAT: Diacylglycerol acyltransferase
DGDG: Digalactosyldiacylglycerol
DGDGS: Digalactoslydiacylglycerol synthase
FFA: Free fatty acid
G3P: Glycerol-3-phosphate
GPAT: Glycerol-3-phosphate acyltransferase
LPA: Lysophosphatidic acid
LPAAT: 2-lysophosphatidic acid acyltransferase
MGDG: Monogalactosyldiacylglycerol
MGDGS: Monogalactosyldiacylglycerol transferase
PA: Phosphatidic acid
PC: Phosphatidylcholine
PP: Phosphatidate phosphatase
PDAT: Phospholipid: diacylglycerol acyltransferase
PE: Phosphatidylethanolamine
TG: Triacylglycerol
TGDG: Trigalactosyldiacylglycerol
