# Supplementary material for: Hypoxia increases triacylglycerol levels and unsaturation in tomato roots
Source: BMC Plant Biol. 2024 Sep 30;24:909. doi: 10.1186/s12870-024-05578-4 (PMC11441241; doi:10.1186/s12870-024-05578-4)
Supplement: Supplementary file 1 — Supplementary Material 1 [file 12870_2024_5578_MOESM1_ESM.docx]

**Hypoxia increases triacylglycerol levels and unsaturation in tomato roots**

Johanna Striesow^1,*^, Marcel Welle^2,*^, Larissa Milena Busch^3^, Sander Bekeschus^1^, Kristian Wende^1^, Christine Stöhr^2^

1 ZIK *plasmatis,* Leibniz Institute for Plasma Science and Technology (INP), Felix-Hausdorff-Str. 2, 17489 Greifswald, Germany

2 Department of Plant Physiology, Greifswald University, Soldmannstr. 15, 17489 Greifswald, Germany

3 Department of Functional Genomics, Greifswald University Medical Center, Felix-Hausdorff-Str. 8, 17489 Greifswald, Germany

* equally contributed as first authors

**Corresponding author:** s-mawell@uni-greifswald.de, kristian.wende@inp-greifswald.de

**Tables**

**Table S1.** **Lipid species found exclusively under hypoxic conditions.** Isobar compounds were detected by their different retention times

| Lipid species | Bulk ID | Chemical formula | Mass  (M-H/M + NH_4_^+^) | Adduct | RT 1 | RT 2 |
| --- | --- | --- | --- | --- | --- | --- |
| PE (16:0_16:4) | PE (30:1) | C37H66NO8P | 682.4453 | -H | 10.91 | 10.10 |
| PE (16:4_18:2) | PE (34:6) | C39H66O8NP | 706.4453 | -H | 9.70 | 8.97 |
| TG (16:0_16:4_16:4) | TG (48:8) | C51H82O6 | 808.6450 | +NH_4_^+^ | 23.68 | 22.34 |
| TG (15:0_16:4_18:3) | TG (49:7) | C52H86O6 | 824.6763 | +NH_4_^+^ | 24.68 | 23.48 |
| TG (15:0_16:4_18:2) | TG (49:6) | C52H88O6 | 826.6919 | +NH_4_^+^ | 25.51 | 24.61 |
| TG (16:3_16:4_18:2) | TG (50:9) | C53H84O6 | 834.6606 | +NH_4_^+^ | 22.86 | 21.49 |
| TG (16:1_16:4_18:3) TG (16:2_16:4_18:2) | TG (50:8) | C53H86O6 | 836.6763 | +NH_4_^+^ | 24.23 | 22.91 |
| TG (16:4_18:2_18:4) | TG (52:10) | C55H86O6 | 860.6763 | +NH_4_^+^ | 23.32 | 22.06 |
| TG (16:4_18:2_18:3) | TG (52:9) | C55H88O6 | 862.6919 | +NH_4_^+^ | 24.49 | 23.26 |
| TG (16:4_18:3_19:2) | TG (53:9) | C56H90O6 | 876.7076 | +NH_4_^+^ | 24.84 | 23.81 |

**RT1/2 = retention time batch 1/2**

**Table S2. Enrichment analysis of lipids from the PCAs first dimension.** Statistical analysis was performed using Fisher´s exact test (*p ≤ 0.05, **p ≤ 0.01, ***p ≤ 0.001).

| Class | Total species number | Species number contributing to separation of conditions | Accumulation | Polarity | p-value |
| --- | --- | --- | --- | --- | --- |
| AcHexSiE | 19 | 0 | Reduced | Positive | **0.032*** |
| DGDG | 27 | 0 | Reduced | Positive | **0.008**** |
| PC | 47 | 3 | Reduced | Positive | **0.029*** |
| TG | 168 | 48 | Enriched | Positive | **0.00010***** |

**Figures**


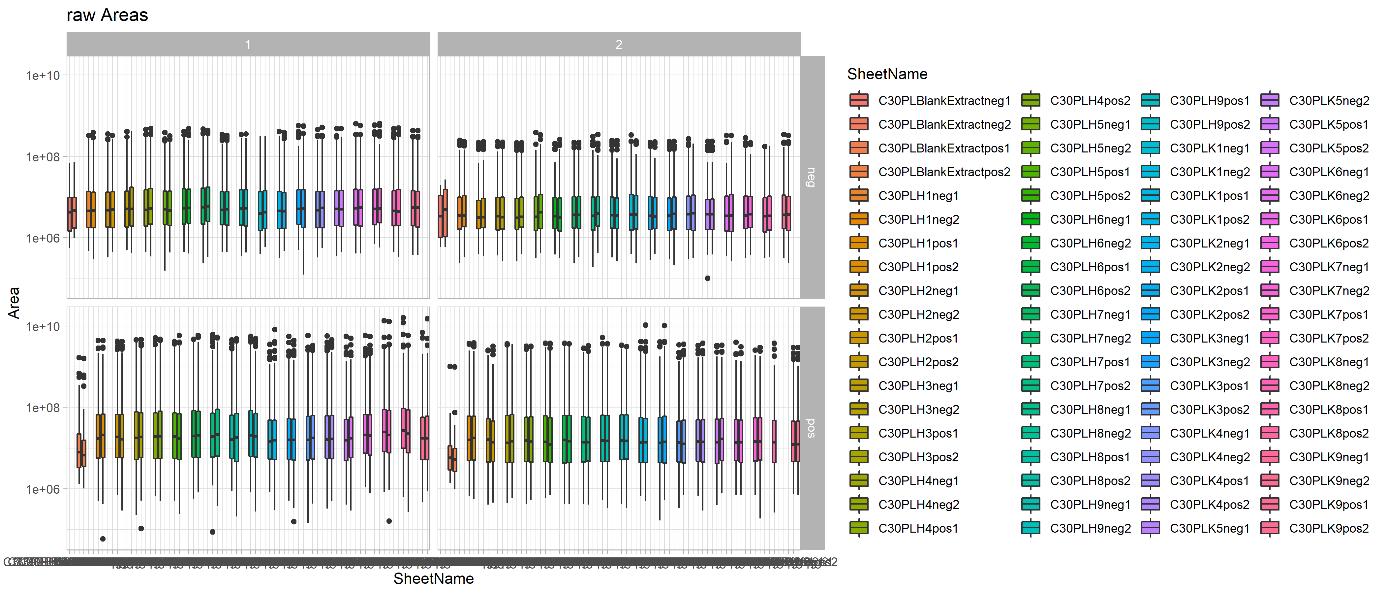


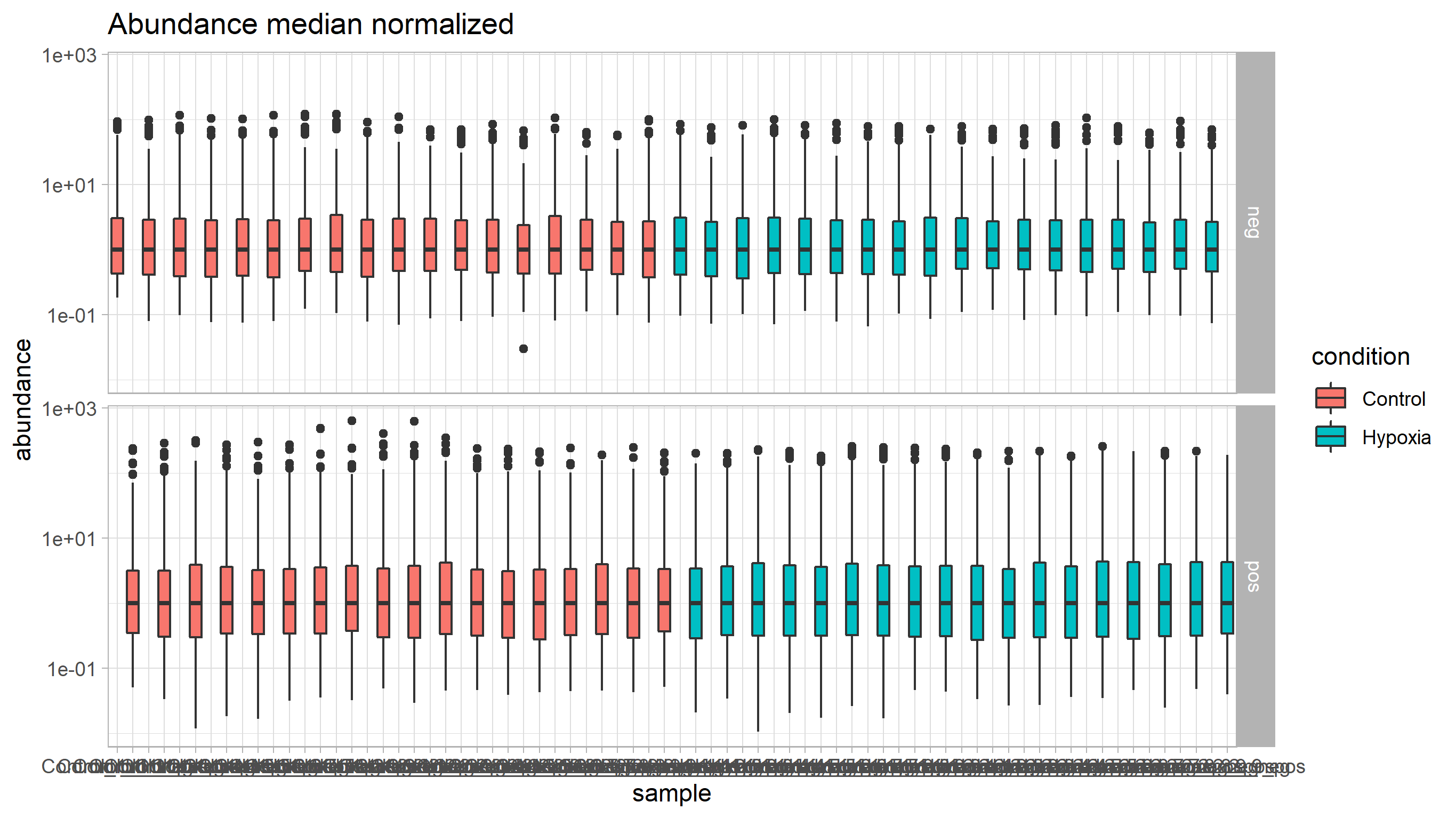


**Figure S1.** Acquired raw data plotted from all samples (**A**). Median-normalized data (**B**). X-axis (represents each individual sample) was removed for simplicity.

**Figure S2: Peak shape and MSMS fragmentation pattern of newly discovered TG species.** Manual inspection of nine TG species exclusively abundant under hypoxic conditions reveals unusual fatty acid 16:4 esterified to TG (**A, B, C, E, F, G, H**).

**Figure S3: DGDG synthesis derived from DG is not affected by hypoxia.**

**Figure S4. PE species esterified with hexadecatetraenoic acid (C16:4) accumulate under hypoxic conditions.** Two PE species were found exclusively in submerged samples (A). Extracted ion chromatograms in positive (B) and negative ionization mode (C) were used for correct retention time annotation of the lipid species. For PE (16:0_16:4), MSMS fragmentation spectra in negative (D) and positive ionization mode (E) were used for the correct annotation of species. In addition, PE (16:4_18:2) fragmentation was annotated in negative (F) and positive ionization mode (G).
